# Immediate and Long-Term Therapy of Patients with Acute Coronary Syndromes with Thienopyridines. Current Status According to the Latest European Society of Cardiology (ESC) Guidelines

**DOI:** 10.5041/RMMJ.10056

**Published:** 2011-07-31

**Authors:** Sigmund Silber

**Affiliations:** Heart Center at the Isar, Munich, Germany

**Keywords:** Acute coronary syndrome, myocardial infarction, clopidogrel, prasugrel, percutaneous coronary intervention, stent

## Abstract

For patients with acute coronary syndrome (ACS), the first priority is to alert emergency services. In addition to an ECG (ideally taken during the first medical contact at the patient’s home), the key of life saving is the immediate antithrombotic therapy with acetylsalicylic acid (ASA) and (unless contraindicated) an injection of unfractionated heparin or bivalirudin as an alternative anticoagulant. Dual anti-platelet therapy (ASA combined with other antiplatelet drugs, like thienopyridines) should be started as soon as possible in the ambulance or at the latest in the hospital. For clopidogrel, a loading dose of 600 mg is the standard. To avoid the risk of an unknown low or missing clopidogrel response, prasugrel is recommended instead, with administration of a loading dose of 60 mg, if no contraindication (s/p stroke or TIA) exists. When PCI is planned, the ambulance must head directly to the nearest hospital with continuous (24/7) PCI service within 90 (to 120) minutes. The maintenance dose for clopidogrel is 75 mg/d; a daily double-dose has not proven to be superior, even in “low responders”. For prasugrel, the maintenance dose is usually 10 mg/d. To avoid bleeding complications in patients ≥ 75 y and/or < 60 kg, a prasugrel maintenance dose of 5 mg/d is recommended. The ESC guidelines recommend DAPT for 1 year after ACS in all patients – independent of the type of ACS and independent of whether any or which coronary stent has been implanted. With DAPT, the patient – and not the stent – is treated.

## BACKGROUND

Despite the extensive scientific knowledge of cardiovascular risk factors and despite educating the general public, cardiovascular diseases continue to be the leading cause of death. Patients with acute coronary syndromes (ACS) are at particular risk. The immediate measures initiated in these patients often determine if the outcome is life or death. The objective of this overview is the evaluation of the current guidelines to effect practical therapy tips for primary and secondary health care providers, with special focus on the antiplatelet treatment with thienopyridines.

## DEFINITION OF ACUTE CORONARY SYNDROME

**Figure 1:**
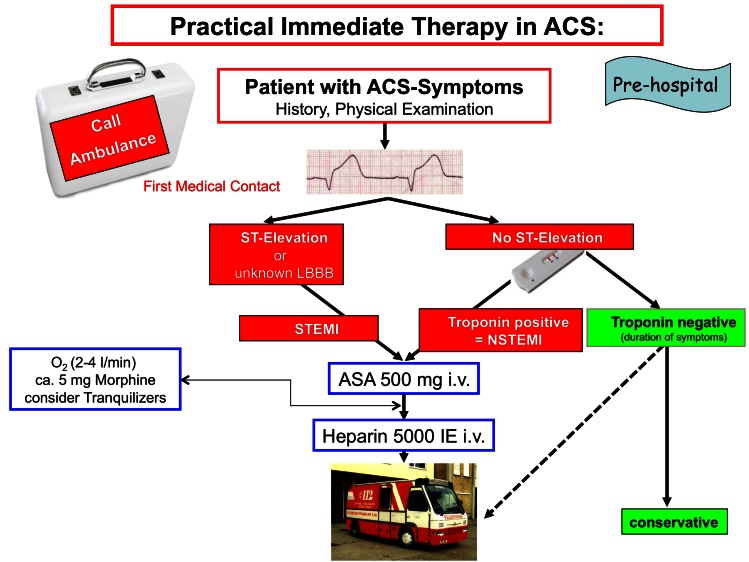
Suggestion for immediate pre-hospital measures in patients with acute coronary syndromes (ACS). Other dosing or oral administration of acetylsalicylic acid (ASA) – within the frame of the new European Society of Cardiology (ESC) guidelines – may also be applied, depending on local practice. (LBBB, left bundle branch block; NSTEMI, non-ST-elevation myocardial infarction; STEMI, ST-segment elevation myocardial infarction.)

Depending on the symptoms and objective findings, ACS comprises three distinct syndromes (Table 1): acute myocardial infarction with persistent ST-segment elevation (STEMI), acute myocardial infarction without ST-segment elevation (NSTEMI), and unstable angina pectoris (UAP). NSTEMI and UAP are often combined to NSTE-ACS. A (presumably) new-onset left bundle branch block (LBBB) is – depending on the symptoms – initially to be regarded as a STEMI. For STEMI, the symptoms and the electrocardiogram (ECG) are sufficient for diagnosis; one does not have to wait for the results of troponin or CK-MB. In an ideal setting with short system delay times, troponin, if determined, would be negative anyway within the first 6 hours after onset of symptoms. For NSTE-ACS, a positive troponin is the first determining factor for NSTEMI (Table 1, Figure 1).

**Table 1:** Definition of the three forms of acute coronary syndromes (ACS).

	**STEMI**	**NSTEMI**	**Unstable angina**
ACS symptoms	+	+	+
ECG	ST-elevation or new LBBB	with or without ST-depression	with or without ST-depression
Troponin	(usually still) negative	positive	negative
Myocardial infarction	yes (based on ST-elevation)	yes (based on troponin)	no

STEMI, ST-segment elevation myocardial infarction; NSTEMI, non-ST-elevation myocardial infarction; LBBB, left bundle branch block.

## PRE-HOSPITAL EMERGENCY MEASURES

The first priority is to alert emergency services – whatever emergency number applies geographically. In addition to general measures including an ECG (ideally taken during the first medical contact at the patient’s home), the key is the immediate antithrombotic therapy: if possible (independent of any history of pre-existing therapy), acetylsalicylic acid (ASA) 500 mg and (unless contraindicated) an injection of 5,000 IU of unfractionated heparin should be immediately administered. Other dosing or oral administration of ASA – within the frame of the new European Society of Cardiology (ESC) guidelines – may also be applied, depending on local practice. If already available at first medical contact, a bolus of bivalirudin can be preferred as an initial alternative to unfractionated heparin (Class IB vs. IC). If the transportation times to the next hospital are short, additional antiplatelet therapy with a thienopyridine can be administered in the hospital. There is no need for “upstream” infusion of glycoprotein IIb/IIIa inhibitors such as abciximab, integrelin, or tirofiban (Class III B).

**Figure 2:**
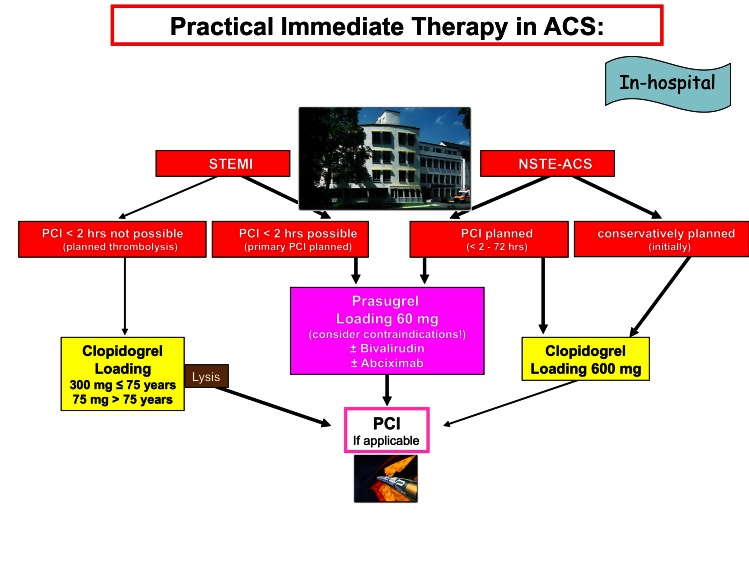
Suggestion for in-hospital therapy of patients with acute coronary syndromes (ACS). If transportation times are too long, then the thienopyridine loading dose should be administered pre-hospital, depending on the planned reperfusion strategy. If thrombolysis is planned, the initial clopidogrel dose depends on age. (PCI, percutaneous coronary intervention; STEMI, ST-segment elevation myocardial infarction; NSTEMI, non-ST-elevation myocardial infarction.)

Of utmost importance is the decision – if possible at a pre-hospital stage – whether a percutaneous coronary intervention (PCI) can or should be performed (Figure 2). When a PCI is planned, the ambulance must head directly to the nearest hospital with continuous (24/7) PCI service (Figure 2) within 90 (to 120) minutes.

## IMMEDIATE MEASURES IN THE HOSPITAL

The basis for optimal oral antithrombotic therapy is a dual antiplatelet therapy (DAPT), which is the combination of ASA with a thienopyridine derivate, i.e. with clopidogrel or prasugrel (ticlopidine is no longer recommended). The data for clopidogrel regarding DAPT for STEMI come mainly from the CLARITY and COMMIT-CCS2 trials (not PCI studies) and regarding NSTE-ACS from the CURE study. These trials were performed using the original clopidogrel hydrogen sulfate. The clinical effect and safety of so-called “generics” (besylate or other compounds) are not established and therefore not recommended.

For prasugrel, the scientific foundation is mainly the TRITON-TIMI 38 trial for all forms of ACS. In this PCI trial the primary combined end-point of cardiovascular death, non-fatal myocardial infarction, and non-fatal stroke was reached in favor of prasugrel (Table 2). On the other hand, the rate of major bleeding was significantly higher with prasugrel as compared to clopidogrel (Table 2). More details, especially the significant reduction of stent thrombosis with prasugrel, are listed in Table 2. Total mortality was relatively low in both groups (Table 2). The advantage of prasugrel was especially pronounced in patients with STEMI (Table 2) or diabetes mellitus (significant reduction of composite primary end-point from 17.0% to 12.2%). In patients with STEMI or diabetes mellitus, this clinical advantage was achieved without a significant difference in major bleeding complications (for STEMI, see Table 2; in diabetic patients it was 2.6% for clopidogrel and 2.5% for prasugrel).

**Table 2:** Results of the randomized TRITON-TIMI 38 study. At 15 months, the primary efficacy end-point, a combination of cardiovascular death, non-fatal myocardial infarction, and non-fatal stroke, was reached in favor of prasugrel. The key safety end-point, non-CABG-related TIMI major bleeding, is also listed (in STEMI not significant). For more details, please see text.

	**Clopidogrel total (*n*= 6795)**	**Prasugrel total (*n*= 6813)**	**Clopidogrel STEMI (*n*= 1765)**	**Prasugrel STEMI (*n*= 1769)**	**Clopidogrel NSTE-ACS (*n*= 5030)**	**Prasugrel NSTE-ACS (*n*= 5044)**
Primary end-point	12.1%	9.9%*	12.4%	10.0%*	12.1%	9.9%*
Total mortality	3.2%	3.0%	4.3%	3.3%	2.4%	2.6%
Non-fatal myocardial infarction	9.5%	7.3%*	9.0%	6.8%*	9.8%	7.5%*
Stent thrombosis	2.4%	1.1%*	2.8	1.6*	2.2%	1.0%*
TIMI major bleeding	1.8%	2.4%*	2.1%	2.4%	1.6%	2.4%*

* *P* < 0.05.

Patients with STEMI – and thus destined for primary PCI – should follow the recent guidelines of the ESC, i.e. DAPT preferably with prasugrel (60 mg loading dose, regardless of age and weight) (Figure 2; level of recommendation for prasugrel = I B, for clopidogrel = I C). The 30-day mortality rate could hereby be significantly reduced: from 2.6% with clopidogrel to 1.6% with prasugrel. For STEMI as well as for NSTE-ACS, prasugrel was able to reduce significantly both the non-fatal myocardial infarction and stent thrombosis (Table 2). Due to the significant increase of fatal bleeding (0.1% versus 0.4%), a history of previous stroke or transient ischemic attack (TIA), however, is a contraindication for prasugrel.

However, in the group of patients with no history of stroke or TIA, age < 75 years, and/or body weight ≥ 60 kg, non- coronary artery bypass graft surgery CABG-related thrombolysis in myocardial infarction (TIMI) major bleeding was no longer significantly different between prasugrel (2.0%) and clopidogrel (1.5%). In this group, the primary efficacy end-point was still significantly reduced with prasugrel (from 11% to 8.3%; *P* < 0.001).

For NSTE-ACS with planned PCI, either prasugrel (IIa B) or clopidogrel (IC) may be administered (Figure 2). Recently, the new USA-guidelines 2011 upgraded prasugrel for planned PCI in NSTE-ACS to a Class IB recommendation.

## LONG-TERM TREATMENT AFTER ACUTE CORONARY SYNDROME

The maintenance dose for clopidogrel is 75 mg/d; a daily double-dose has not proven to be superior, even in “low responders”. For prasugrel, the maintenance dose is usually 10 mg/d. To avoid bleeding complications in patients ≥ 75 y and/or < 60 kg, a prasugrel maintenance dose of 5 mg/d is recommended. Since the efficacy of prasugrel is independent of genetic factors, a genetic test or *in-vitro* platelet function test for prasugrel is not necessary. A possible interaction of proton pump inhibitors (PPI) with clopidogrel is still debated, but prasugrel seems to be independent of this postulated interaction. The ESC guidelines re-commend DAPT for 1 year after ACS in all patients – independent of the type of ACS and independent of whether any or which coronary stent has been implanted. With DAPT, the patient – and not the stent – is treated.

## Abbreviations:

ACS: acute coronary syndromes;
ASA: acetylsalicylic acid;
DAPT: dual antiplatelet therapy;
ECG: electrocardiogram;
ESC: European Society of Cardiology;
NSTE-ACS: non-ST-elevation acute coronary syndrome;
NSTEMI: non-ST-elevation myocardial infarction;
PCI: percutaneous coronary intervention;
STEMI: ST-segment elevation myocardial infarction;
TIA: transient ischemic attack;
UAP: unstable angina pectoris.
